# Blockade of intercostobrachial nerve by an erector spinae plane block at T2 level: a case report

**DOI:** 10.1186/s40981-023-00641-9

**Published:** 2023-08-04

**Authors:** Takayuki Yoshida, Tatsuo Nakamoto

**Affiliations:** Department of Anesthesiology, Kansai Medical University Medical Center, 10-15 Fumizono-Cho, Moriguchi City, Osaka, 570-8507 Japan

**Keywords:** Brachial plexus block, Brachial vein transposition arteriovenous fistula, Erector spinae plane block, Intercostobrachial nerve, Lateral cutaneous branch, Paravertebral block, Thoracic wall block

## Abstract

**Background:**

The intercostobrachial nerve blockade is required, in addition to brachial plexus block, to anesthetize the entire upper arm. No studies have described the use of erector spinae plane (ESP) block for an intercostobrachial nerve block.

**Case presentation:**

A 72-year-old man was scheduled to undergo left brachial vein transposition-arteriovenous fistula creation for hemodialysis access. An ultrasound-guided infraclavicular brachial plexus block was performed using a mixture of 0.5% levobupivacaine (12.5 ml) and 2% lidocaine (12.5 ml). An ESP block was implemented using 10 ml of the same local anesthetic at the T2 level. A pinprick test showed that the entire upper arm and lateral aspect of the left upper chest wall were anesthetized 20 min after the blocks. Surgery was successfully performed without the need for general anesthesia.

**Conclusions:**

In the present case, an ESP block performed at the T2 level provided sensory loss of the area innervated by the intercostobrachial nerve.

## Background

The axillary, radial, medial brachial cutaneous, medial antebrachial cutaneous, and intercostobrachial nerves innervate and provide sensation to the upper arm [1]. Except for the intercostobrachial nerve, the other nerves can be blocked by supraclavicular and infraclavicular approaches for brachial plexus blocks [2, 3]. Consequently, to ensure anesthesia of the entire upper arm using peripheral nerve blocks, an additional procedure is needed to block the intercostobrachial nerve, which provides sensory innervation to the skin of the axilla, the medial part of the upper arm, and the lateral side of the thoracic wall close to the axilla [4, 5].

The intercostobrachial nerve is the lateral cutaneous branch of the ventral ramus of the second thoracic spinal nerve [5]. Blockade of the intercostobrachial nerve can be achieved by ultrasound-guided local anesthetic infiltration in the axilla [4] or in the serratus plane [6–8]. Paravertebral blocks performed at high thoracic levels also involve the second thoracic spinal nerve. Recently, an erector spinae plane (ESP) block has gained popularity as an alternative to paravertebral blocks, as it is theoretically safer [9]. Nevertheless, the feasibility of blocking the ventral rami of spinal nerves using ESP blocks remains controversial [10].

Herein, we describe a case of brachial vein transposition-arteriovenous fistula creation managed using a combination of an ESP block at the level of second thoracic vertebra (T2), an infraclavicular brachial plexus block, and sedation.

## Case presentation

Consent for publication was obtained from the patient for reporting case details.

A 72-year-old man (height 165.5 cm, weight 72.2 kg) was scheduled for left brachial vein transposition-arteriovenous fistula creation for hemodialysis access. The patient had a history of myocardial infarction, diagnosed at 52 years of age. One month prior, he developed acute myocardial infarction requiring the insertion of a stent in the left anterior descending coronary artery. Echocardiography performed after coronary stenting revealed a left ventricular ejection fraction of 27%. The patient subsequently developed chronic renal failure, which was attributed to a history of heart failure and use of contrast dye for coronary angiography; he underwent hemodialysis following catheterization of the left femoral vein for vascular access. After coronary stenting, the patient started the intake of aspirin 100 mg and clopidogrel 75 mg. Clopidogrel was withheld for 14 days prior to the surgery, while aspirin was continued. For maintenance hemodialysis with brachial vein transposition-arteriovenous fistula, the surgeon planned an incision extending from a few centimeters proximal to the level of the antecubital fossa toward the axilla by approximately 15 cm on the medial aspect of the left upper arm. Considering the risk of perioperative heart failure and to avoid general anesthesia, the patient was given regional anesthesia combined with sedation.

**Fig. 1:**
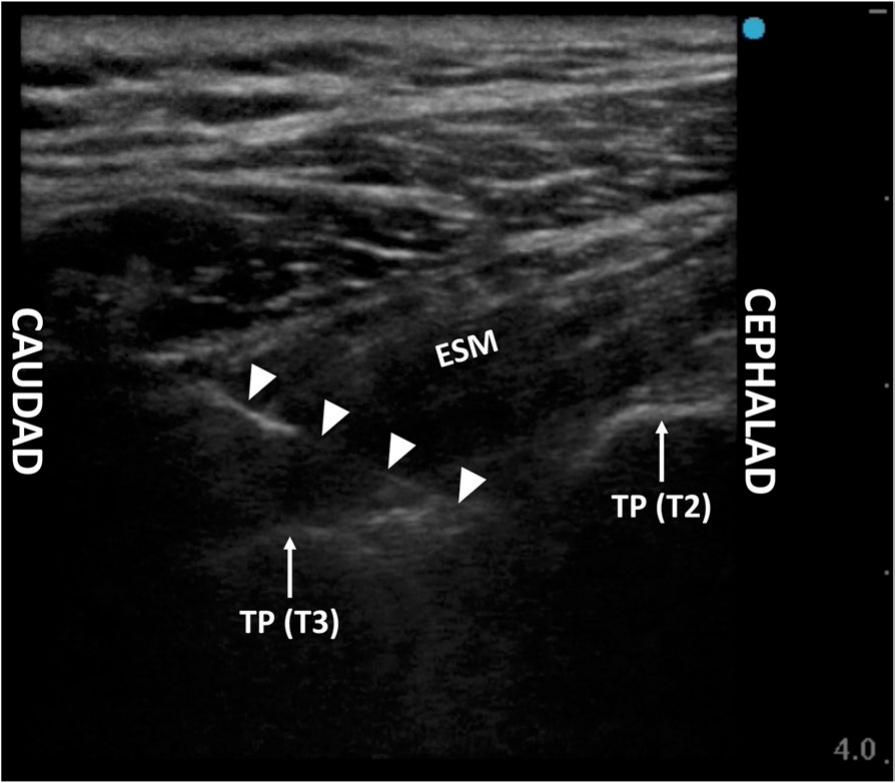
Ultrasound image of erector spinae plane block performed at the intertransverse processes space between the T2 and T3. The triangles indicate the block needle. ESM, erector spinae muscles; TP, transverse process

Standard monitoring, including electrocardiography (ECG), pulse oximetry, and noninvasive blood pressure monitoring, was established on the patient’s arrival in the operating room. Subsequently, 2 mg of intravenous midazolam was administered for anxiolytic purposes. Throughout the block and during the surgical procedure, 5 l/min of oxygen was delivered via a face mask. An ultrasound-guided infraclavicular brachial plexus block was performed using a mixture of 12.5 ml each of 0.5% levobupivacaine and 2% lidocaine, with the patient in the supine position. A linear transducer (HFL38xi; FUJIFILM SonoSite Inc., Tokyo, Japan) in a sterile cover connected to an ultrasound apparatus (S II; FUJIFILM SonoSite Inc.) was placed immediately medial to the left coracoid process, inferior to the clavicle, in the sagittal plane to visualize the medial, lateral, and posterior cords of the brachial plexus. A 22-gauge echogenic block needle (Uniever; Unisis, Tokyo, Japan) was introduced superior to the cranial end of the transducer and advanced in-plane with the transducer toward the cords of the brachial plexus. Next, 10, 10, and 5 ml of the local anesthetic mixture were injected around the posterior, lateral, and medial cords, respectively. Subsequently, the patient was placed in the right lateral decubitus position. A linear transducer was placed on the superoposterior aspect of the left shoulder close to the neck to observe the short-axis view of the transverse processes of T2 and T3. The same type of block needle was inserted and advanced in a caudad-to-cephalad direction toward the space between the two transverse processes using in-plane ultrasound guidance. Immediately after penetrating the anterior fascia of the erector spinae muscles, 10 ml of the same local anesthetic mixture was administered (Fig. 1). Twenty minutes after the completion of the nerve blocks, a pinprick test showed that the entire upper arm and lateral aspect of the left upper chest wall were anesthetized. Thereafter, surgery was initiated.

**Fig. 2:**
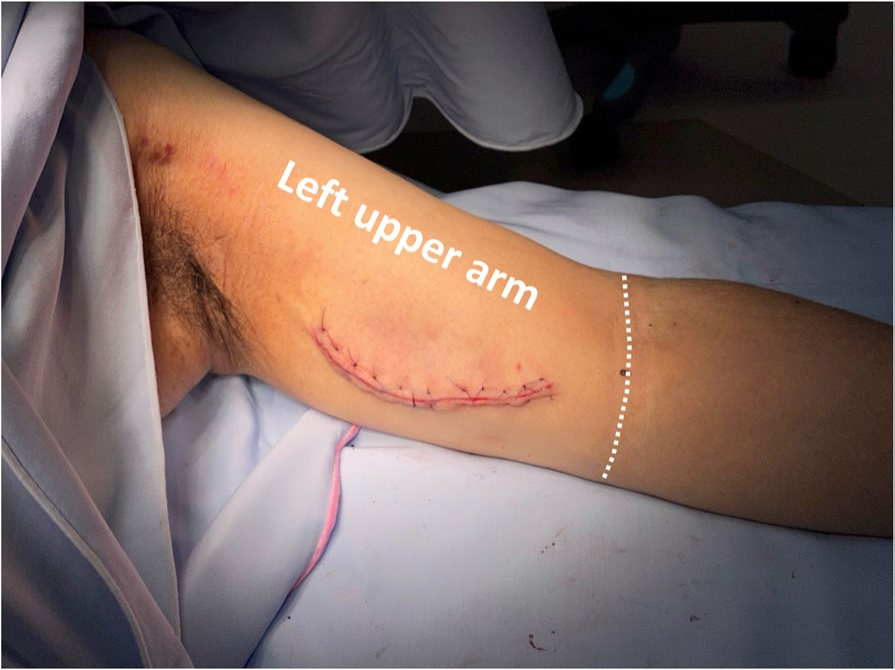
Incision for brachial vein transposition-arteriovenous fistula creation on the left upper arm. The dotted line indicates the cubital fossa

Sedation was provided with a target-controlled infusion of propofol at 1.2 to 1.4 µg/ml during the surgical procedure. No additional analgesics or anesthetics were administered. The patient did not complain of any pain during surgery (Fig. 2). Systolic and diastolic blood pressures fluctuated from 95 to 115 mmHg and 55 to 65 mmHg, respectively. The patient’s heart rate was stable at approximately 70 bpm during surgery, which took 141 min.

Twenty minutes after surgery and 3 h after the blocks were performed, a pinprick test showed complete sensory loss on the entire left upper limb, including the areas innervated by the intercostobrachial and medial brachial cutaneous nerves and the left lateral side of the upper thoracic wall. Complete motor block was observed in the left elbow, wrist, and finger joints. At 5.5 h after the block, return of sensation in the axilla and the left lateral side of the thoracic wall was confirmed by pinprick, while sensory loss in the other areas and motor block persisted. Approximately 18.5 h after completion of the nerve blocks, the patient started feeling pain at the incision site.

## Discussion

In the present case, a combination of infraclavicular brachial plexus block and ESP block at the T2 level anesthetized the entire upper arm, including the area innervated by the intercostobrachial nerve, providing successful anesthetic management for brachial vein transposition-arteriovenous fistula creation without general anesthesia.

The medial cord of the brachial plexus gives off the medial brachial and antebrachial cutaneous nerves, while the posterior cord gives rise to the axillary nerve. Therefore, brachial plexus block approaches performed at or proximal to the level of these cords can block these cutaneous nerves as well as the axillary and radial nerves [2, 3], but not the intercostobrachial nerve, i.e., the lateral cutaneous branch of the ventral ramus of the T2 spinal nerve.

Thoracic wall blocks administered immediately superficial or deep to the serratus anterior muscle target the lateral cutaneous branches of thoracic spinal nerves [6–8]. Hence, serratus plane blocks performed at the second or adjacent rib can produce an intercostobrachial nerve blockade. Indeed, Moustafa and Kandeel [8] showed that to block the intercostobrachial nerve, injecting between the pectoralis minor and serratus anterior muscles at the level of the third rib in the anterior axillary line provided a significantly higher success rate compared with subcutaneous injection along the medial side of the upper arm. Furthermore, ultrasound-guided local anesthetic infiltration in the proximity of the medial brachial cutaneous and intercostobrachial nerves on the surface of the latissimus dorsi muscle at the axilla has been reported with a success rate of 92.9% [4]. However, inadvertent vessel puncture at the axilla might reduce blood flow to the brachial vein transposition-arteriovenous fistula. Bearing this in mind, we chose to perform an ESP block to obtain an intercostobrachial nerve blockade, although no article has described the use of ESP blocks for this purpose.

Some studies have reported that an ESP block involves not only the dorsal rami of the thoracic spinal nerves but also the ventral rami [10–12]. Nevertheless, the likelihood of anterior spread (i.e., the ventral rami involvement) seen with the ESP block has been challenged [10, 13, 14]. Cadaver evaluations and MRI studies in healthy volunteers have shown that ESP injection spreads dye and local anesthetic over multiple paravertebral and epidural segments [10, 15–18]. In addition, some clinical studies have utilized ESP blocks for analgesia after breast and abdominal surgery and have reported promising results [11, 12]. In contrast, another cadaver study by Ivanusic et al. [19] denied the paravertebral and epidural spread of ESP blocks. They demonstrated that the local anesthetic injected into the ESP spreads laterally and reaches the lateral cutaneous branch of the ventral spinal nerve. Even this lateral spread along the lateral cutaneous branch can block the intercostobrachial nerve, which is the lateral cutaneous branch of the second intercostal nerve. Contrastingly, most studies investigating ESP injection spread have reported that lateral spread is limited by the lateral boundary of the erector spinae muscles, thereby contradicting Ivanusic’s finding [10]. Recently, Harbell et al. [16] used cadavers and reported that ESP injection, administered *between* the transverse processes, and not *on* the posterior surface of the transverse process, would be the key to attaining involvement of the ventral rami in the paravertebral space. Considering this, we administered a local anesthetic into the ESP between the T2 and T3 transverse processes, resulting in successful blockade of the intercostobrachial nerve, which implied the anterior spread of the local anesthetic by the ESP block.

In the present case, we injected only 10 ml of local anesthetic into the ESP because our goal was to anesthetize the area innervated by the intercostobrachial nerve, while the blockade of the T2 spinal nerve was adequate. Therefore, we did not assess the entire distribution of sensory loss on the thoracic wall.

In conclusion, we propose that an ESP block performed between the transverse processes of T2 and T3 causes sensory loss in the area innervated by the intercostobrachial nerve and is helpful for anesthesia management for upper limb surgeries.

## Data Availability

Not applicable due to patient privacy concerns.

## Abbreviations

ECG: Electrocardiogram
ESP: Erector spinae plane
MRI: Magnetic resonance imaging
